# Feud or Friend? The Role of the *miR-17-92* Cluster in Tumorigenesis

**DOI:** 10.2174/138920210790886853

**Published:** 2010-04

**Authors:** Jie Xiang, Ji Wu

**Affiliations:** School of Life Sciences and Biotechnology, Shanghai Jiao Tong University, Shanghai, 200240, P.R. China

**Keywords:** microRNAs (miRNAs), the *miR-17-92* cluster, oncomir, cancer, siRNAs, c-Myc, E2Fs.

## Abstract

MicroRNAs (miRNAs) are short, noncoding, and single-stranded RNA molecules that negatively regulate gene expression. They are evolutionarily conserved from plants to animals. During the last decade, miRNAs have been demonstrated as regulators in fundamental biological processes, including cell growth, proliferation, differentiation and apoptosis. By base pairing to the complementary sites in the mRNA of the target gene, miRNA can lead to repression of protein translation or cleavage of mRNA. Among over 700 miRNAs identified in the human genome, several of them were confirmed as ‘oncomirs’, which denote miRNAs associated with initiation and progression of cancers. Generally, depending on their target genes, these miRNAs function as tumor suppressors or oncogenes. However, the *miR-17-92 *cluster in the human genome, which encodes 7 mature microRNAs, has been validated as regulator showing both oncogenic and tumor suppressive properties. The *miR-17-92 *cluster targets mRNAs involved in distinct pathways so that it may exert opposing effects. The transcription factors E2Fs and c-Myc, which play critical roles in tumorigenesis, could interact with the cluster. The feedback loops, which are comprised of the transcription factors and the miR-17-92 cluster, weave a complex regulation net work of cancers. The duality of this cluster reflects the complexities of cancer progressions as well as the intricacies of the regulation network of miRNAs and their targets. With the help of the development of new experimental methods and bioinformatics, further researches on the* miR-17-92* cluster and other oncomirs will give new insights into cancer diagnosis, therapy, and prognosis.

## INTRODUCTION

MicroRNAs (miRNAs) are a family of endogenous, non-coding, and single-stranded RNA molecules of 17-~27-nucleotides in length that regulate gene expression posttranscriptionally [1]. The first miRNA, *lin-4*, was identified in 1993 [2]. Ambros and colleagues confirmed that using an antisense complemental mechanism, a novel 22-nucleotide small RNA encoded by *lin-4* repressed the expression of lin-14 protein. This downregulation led to the change of temporal pattern formation in* Caenorhabditis elegans* [3]*.* This finding was considered as a genetic oddity in *C. elegans*, therefore, little attention was paid to this small RNA. In 2000, Reinhart *et al.* discovered the second miRNA, *let-7*, which was also involved in *C. elegans* development [4]. Researchers found that the sequence and temporal expression of* let-7 *were conserved in a wide range of animal species, including vertebrate [5]. The “novelty” soon became the “harbinger”. Since then, thousands of miRNAs have been identified in various species, such as plants [6], worms [7-10], flies [11, 12] and mammals [13, 14].

Owing to the development of biochemical searches and informatic analysis during the last decade, researchers around the world have published numerous stories in high-profile magazines to unveil the big roles played by these tiny molecules. The explosion of miRNAs research has propelled the discoveries that miRNAs are of great importance in fundamental biological processes, including development [15, 16, 17], cellular growth, proliferation, apoptosis, glucose and lipid metabolism [18], viral infection [19], insulin secretion [20] and immune response [21]. To date, in the human genome, 721 miRNAs have been annotated in the miRBase (http://microrna.sanger.ac.uk/sequences/). However, they do not include the whole human miRNAome, it is estimated that the final number is probably over 1,000. [22, 23, 24]

As miRNAs play pivotal roles in multiple biological processes, aberrant expression of this family of regulators might result in human diseases. In this review, we mainly focus on the association between miRNAs and cancers, the role of the *miR-17-92* cluster in tumorigenesis, and potential applications of miRNAs in cancer diagnosis, therapy and prognosis.

## MicroRNAs BIOGENESIS

The biogenesis pathway of miRNAs in animals was elucidated by Bartel *et al*. in 2004 [16]. Fig. (**1**) shows the pathway briefly. Some microRNAs come from distinct transcription units, while some other microRNA genes are clustered in the genome [16]. Usually, there are two or three miRNA genes in a cluster, however, larger clusters including more miRNA genes have also been identified. For example, the *miR-17-92* cluster, whose function will be elaborated in the following paragraphs, is composed of seven miRNA genes [25]. MiRNA is transcribed by RNA polymerase II in nucleus, this early large transcript termed pri-miRNA is capped and polyadenylated. Then the pri-miRNA is processed by the Drosha RNase III endonuclease, and the cleavage usually liberates the pre-miRNA, which is ~75-nucleotides in length with stem loop. This step is critical and a double-stranded-RNA-binding protein, called DGCR8 in mammals, is needed for processing [26]. Sequentially, the pre-miRNA is exported to the cytoplasm by Ran-GTP and the transporter Exportin-5. In the cytoplasm, pre-miRNA is processed by the ribonuclease Dicer, which is also RNase III endonuclease and plays a similar role in the biogenesis of short interfering RNAs (siRNAs). Dicer cuts both strands of the precursor, leaving a double-stranded (ds) RNA molecule of approximately 22-nucleotides in length. The molecule is called the miRNA:miRNA* duplex and is composed of mature miRNA strand and the sequences derived from the opposing arm. The duplex is a transient intermediate during the biogenesis. After processing, the active strand of the duplex assembles into the RNA-induced silencing complex (RISC), while the miRNA*, which is the inactive strand, is ejected and degraded. The mature miRNA incorporated into the RISC binds to the complementary sites in the mRNA of the target gene. Argonaute (Ago) proteins are indispensable components of the RISC and play critical part in the post-transcriptional gene silencing (PTGS). The complementarity of the miRNA and the mRNA usually leads to mRNA cleavage or translational repression. Perfect or near-perfect complementarity is required for mRNA cleavage, whereas the lower degree of complementarity results in translational repression. In the animals, the latter mechanism is more prevalent.

SiRNAs are a class of double-stranded RNAs originated from long and perfectly base-paired dsRNA, which can also direct PTGS. Since Fire and Mello discovered this gene silencing pathway in 1998 [27], siRNAs have been intriguing not only as an efficient experimental tool to uncover the gene function but also as a potential remedy for diseases. Although miRNAs and siRNAs share similar molecular characteristics, biogenesis and functions [28], there are some noticeable distinctions between these two families of small RNAs [16, 26]. Besides the differences in biogenesis pathways, siRNAs silence almost exclusively the same locus from which they are produced (auto-silencing), whereas miRNAs silence genes different from which their primary transcripts originate (hetero-silencing). In related organisms, the degree of the sequence conservation of miRNAs is greater than that of siRNAs. SiRNA-mediated gene silencing represents an evolutionarily conserved method of genome defense against exogenous nucleic acids, such as virus, transgenes and transposons [29]. However, as described earlier, miRNAs function as essential regulators in fundamental biological processes in organisms.

## ONCOMIRS AND TUMORIGENESIS

Recent evidences indicate that miRNAs can function as tumor suppressors and oncogenes, and these miRNAs are referred to as ‘oncomirs’ [17, 30, 31]. When cells are damaged, uncontroled proliferation and inappropriate survival of these cells might lead to cancer. Oncogenes and tumor-suppressor genes are two pivotal factors in tumorigenesis. In normal tissue, proper regulation of miRNAs maintains a normal rate of development, cell growth, proliferation, differentiation and apoptosis. However, deregulation of miRNAs target mRNAs of cancer-associated genes probably put cell in danger of canceration. If the target gene is an oncogene, the loss of the miRNA, which functions as a tumor suppressor, might lead to a high expression level of the oncoprotein, therefore tumor formation is likely to happen. On the other hand, as to miRNA functions as an oncogene, constitutively amplification or overexpression of this miRNA could cause immoderate repression of its target gene, which has a role of tumor suppressor gene, thus, in this situation, cell is likely to confront the threat of tumorigenesis [30]. Using genome-wide mapping and northern blotting, researchers demonstrated that microRNA genes are frequently located at fragile sites, minimal regions of loss of heterozygosity, minimal regions of amplification (minimal amplicons) or other cancer-associated genomic regions [25]. Furthermore, several research groups reported deregulations of miRNAs in cancer samples. *MiR-15* and *miR-16* are downregulated in 68% of chronic lymphocytic leukemia patients [32]. Michael and colleagues found that *miR-143* and *miR-145* display reduced steady-state levels of the mature miRNA at the adenomatous and cancer stages of colorectal neoplasia [33]. Reduced expression of *let-7* in lung cancers has also been observed [33, 34]. Besides, evidences reveal microRNAs function as tumor suppressors, studies also show that some miRNAs play roles as oncogenes. For example, Chan *et al. *reported overexpression of *miR-21* in glioblastoma tumor tissues and cell lines in human [35]. Deregulations of the* miR-17-92 *in different cancer samples have been reported frequently, and studies implicated that this cluster performs activity of oncogene as well as tumor suppressor. Thus, wide interest has been attracted to this especial microRNA cluster.

## THE* miR-17-92* CLUSTER AND TUMORIGENESIS

A polycistronic microRNA cluster termed *miR-17-92*, located in *chromosome 13 open reading frame 25 *(*C13orf25*) in the human genome, encodes seven miRNAs: *miR-17-5p*, *miR-17-3p*, *miR-18a*, *miR-19a*, *miR-20a*, *miR-19b *and *miR-92-1.* Fig. (**2**) shows the genomic organization of this cluster.

### The Oncogenic Role of the *miR-17-92* Cluster

The amplification of 13q31-q32, which is the locus of the *miR-17-92 *cluster, have been reported in hematopoietic malignancies and other solid tumors [36], including diffuse large B-cell lymphoma (DLBCL), mantle cell lymphoma, follicular lymphoma, primary cutaneous B-cell lymphoma, nasal-type natural killer/T-cell lymphoma, glioma, non-small cell lung cancer, bladder cancer, squamous-cell carcinoma of the head and neck, peripheral nerve sheath tumor, malignant fibrous histiocytoma, alveolar rhabdomyosarcoma, liposarcoma and colon carcinomas [36, 37, 38]. *C13orf25* has been implicated by Ota* et al. *as the gene for the amplification of 13q31-q32 in tumors [36]. Later, He and colleagues [39] observed significant overexpression of pri-*miR-17-92* in 65% of human tumor samples. Five miRNAs of the cluster (*miR-92-1*,* miR-19a*, *miR-20a*, *miR-19b*, *miR-17-5p*) were upregulated in these cancer cell lines. To further test the role of the *miR-17-92 *cluster in tumorigenesis, they turned to a mouse B-cell lymphoma model. Enforced expression of the cluster accelerated the progression of malignant lymphomas in mice. Results indicate that the *miR-17-92* cluster functions as an oncogene in a cooperative way acted with c-Myc, which is an oncogenic transcription factor often mutated or amplified in human cancers. Dysregulated expression of this oncoprotein in human malignancy has been reported as one of the most common abnormalities [40]. Previous researches have shown that c-Myc could regulate cell growth by inducing both cell proliferation and apoptosis [41]. As an absence of apoptosis was observed in the lymphomas derived from mice expressing both c-myc and miR-17-19b (a subset of the miR-17-92 cluster), the miR-17-92 cluster possibly targets apoptotic factors that are activated, when c-Myc is overexpressed [39]. Altogether, data from He *et al*. suggest an oncogenic role of miR-17-92 cluster and the researchers named the primary transcript for these miRNAs ‘OncomiR-1’ [39]. Therefore, the term ‘oncomir’ was coined to denote microRNA involved in the initiation and progression of cancers. Several members of the cluster are overexpressed separately and simultaneously in human solid tumors, including cancers of breast, colon, lung, stomach, pancreas and prostate [38]. Using chromatin immunoprecipitation (ChIP) assays, O’Donnell *et al. *confirmed that human c-Myc binds directly to the *miR-17-92* cluster genomic locus on chromosome 13, providing strong evidence that the transcription of this cluster is directly induced by c-Myc [42]. E2F family of transcription factors, including E2F1, E2F2 and E2F3, have been reported to drive progression from G_1_ into S phase in mammalian cells by activating expression of genes involved in DNA replication and cell cycle control. Moreover, it has been validated that high levels of E2Fs, E2F1 in particular, can induce apoptosis in response to DNA damage [43]. The research group reported that E2F1 is negatively regulated by *miR-17-5p* and *miR-20a*. Since previous studies have suggested that c-Myc and members of E2Fs can activate one another’s transcription, a tightly controled network is established, through which c-Myc could activate the E2F1 transcription and limit its translation simultaneously [42]. Furthermore, several lines of evidence verified that the E2F transcription factors could induce the expression of miRNAs of the cluster, and two other members of E2Fs, E2F2 and E2F3 are targeted by the *miR-17-92 *cluster [44, 45]. Collectively, a complex interaction network comprising of c-Myc, E2Fs and the *miR-17-92* cluster is unraveled (Fig. (**3**)). In this regulatory network model, microRNAs of the cluster downregulate the E2Fs, as a result, the proapoptotic role of E2Fs is inhibited. Therefore, the *miR-17-92* cluster exerts its oncogenic function through assisting the DNA-damaged cell in escaping the fate of programmed cell death.

Besides E2Fs, several other negative regulators of the G_1_-S checkpoint or proapoptotic proteins are targeted by the *miR-17-92 *cluster. The expression of cyclin-dependent kinase inhibitor CDKN1A (p21) is inhibited by *miR-17-5p*, *miR-18a*, and *miR-20a*. Another member of the *miR-17-92* cluster, *miR-92-1 *could repress the expression level of the tumor suppressor gene, *BIM*, which has a role of proapoptosis [46, 47]. More recently, researchers observed that *miR-17-92 *cluster could counterbalance the generation of DNA damage in RB-inactivated small-cell lung cancer (SCLC), which leads to genetic instability in this type of malignancy [48]. The action of microRNAs in the process of angiogenesis, which is essential for tumor development and metastasis, should not be neglected. Dews *et al*. demonstrated that c-Myc functions as an inducer of angiogenesis in solid tumors through activating the transcription of the miR-17-92 cluster, which downregulates anti-angiogenic proteins such as thrombospondin-1 (Tsp1) and connective tissue growth factor (CTGF) [49, 50]. The miR-17-92 cluster is highly expressed in mouse ES cells and chicken embryos, while the expression level of the cluster decreases in differentiated tissues [51, 52]. Loss of differentiation and uncontrollable proliferation are two major characteristics of cancer, and mounting evidence indicates that cancer stem cell model is responsible for cancerogenesis [53], thus, the “stemness” of this cluster suggests a correlation between high level expression of the *miR-17-92 *cluster in cancer samples and continuous self-renewal of cancer stem cells [54].

### The Tumor Suppressive Role of the miR-17-92 Cluster

Accumulating evidences indicate the oncogenic role of the* miR-17-92 *cluster, paradoxically, researchers observed that the cluster could act as a tumor suppressor in some circumstances. Loss of heterozygosity at 13q12-q13 is associated with multiple tumor progression and poor prognosis, including breast cancer, squamous cell carcinoma of the larynx, retinoblastoma, hepatocellular carcinoma and nasopharyngeal carcinoma [55]. Using a high-resolution array-based comparative genomic hybridization in human tumor specimens, Zhang *et al.* observed the* miR-17-92 *cluster was deleted in 16.5% of ovarian cancers, 21.9% of breast cancers, and 20.0% of melanomas [56]. As described earlier, c-Myc induces expression of the E2Fs, which drive progression from G_1_ into S phase in cells. In this circumstance, the transition promotes the proliferation of cells, so that E2Fs functions as oncogenic proteins. Thus, the *miR-17-92 *cluster, which downregulates E2Fs, may serve as a brake on excessive proliferation. As a consequence, the cluster performs the anti-tumorigenic activity. The *miR-17-5p* exerts its role of tumor suppressor in breast cancer cells by repressing the expression of *AIB1* (named for “amplified in breast cancer 1”). Overexpression or downregulation of the *miR-17-5p* could suppress or promote breast cancer cell proliferation, respectively [57]. Recently, Yu *et al. *reported that Cyclin D1, which has been demonstrated as an oncogenic protein in breast cancer cell, is negatively regulated by *miR-17-5p* and *miR-20a* [58].

### Clues to Unravel the Paradox

Table **1**. summarizes functions of the targets of the miR-17-92 cluster mentioned above. The fact that *miR-17-92* cluster functions both as oncogenes and tumor suppressors, implicates the complexities of cancer progressions as well as the intricacies of the regulation network of miRNAs. A single miRNA could target hundreds of mRNAs involved in distinct pathways, thus it may perform opposing functions. By using web-based Ingenuity Pathways Analysis (IPA), Cloonan and colleagues uncovered a large genetic network in which the* miR-17-5p* is a key regulator of the G_1_/S phase cell cycle transition [59]. More than 20 genes involved in the transition between the G_1_/S phase are directly targeted by this single microRNA. *MiR-17-5p* can exert both oncogenic and tumor suppressive function through decreasing the expression levels of anti-proliferative genes and pro-proliferative genes, respectively. These findings reflect that a single miRNA acts opposing roles in order to maintain equilibrium *in vivo*. Perhaps the role of the *miR-17-92 *cluster as oncogene or tumor suppressor is dependent on the cell type and the expression pattern and levels of the target mRNAs [59]. Besides target mRNAs profile, epigenetic regulation and abnormalities in miRNA-processing genes and proteins are two important influential factors for miRNAs expression [60]. Several groups have verified that DNA hypomethylation, CpG island hypermethylation and histone-modification could affect miRNA expression. As microRNAs biogenesis is essential for the expression levels of miRNAs, alterations in the miRNA processing machinery definitely will result in dramatic effects on miRNAs expression. Essential proteins take part in miRNAs biogenesis, such as Drosha and Dicer, may contribute to aberrant miRNAs expression [60].

## POTENTIAL USES OF THE ONCOMIRS IN CANCER DIAGNOSIS, THERAPY AND PROGNOSIS

The most commonly used high-throughput technique for miRNA profiling is oligonucleotide miRNA microarray analysis. Liu *et al.* firstly reported the process for genome-wide miRNA profiling in human and mouse tissues [61]. Lu *et al.* reported that a bead-based flow cytometric miRNA expression profiling method could accurately reflect the developmental lineage and differentiation state of the tumors [62]. Based on the use of locked nucleic acid (LNA)-modified oligonucleotides, researchers developed a microarray platform (miChip) displays superior sensitivity [63]. These researches are crucial in providing novel insights into cancer diagnosis using microRNA profiling. Taking advantage of these high-throughput platforms, miRNA expression profiling have been widely studied in human cancers, including solid tumors and hematopoietic malignancies [64].

The discovery of oncomirs is likely to introduce novel approaches using miRNA therapy to treat cancers. Designing antisense oligonucleotides that are complementary to mature oncogenic miRNAs might inactivate the miRNAs and lead to inhibition of tumor growth. These synthetic oligonucleotides are termed as anti-miRNA oligonucleotides (AMOs). Matsubara* et al. *reported that using AMOs, they successfully induced apoptosis selectively in lung cancer cells overexpressing miRNAs of the *miR-17-92* cluster [65]. Krützfeldt *et al*. reported antagomirs, which are chemically engineered oligonucleotides, are powerful tools to silence specific miRNAs *in vivo* [66]. These findings might ultimately lead to future translation into clinical applications. On the other hand, as to oncomirs function as tumor suppressors, techniques to overexpress these miRNAs could be used to treat certain tumour types. These miRNAs could be delivered by virus or liposomes of transient expression systems [31].

Takamizawa *et al*. identified that reduced expression of the *let-7* microRNAs in human lung cancers is associated with shortened postoperative survival [34]. Yanaihara *et al*. found high expression of *miR-155* and low expression of *let-7a-2* are correlated with poor survival in lung adenocarcinomas [67]. Prognostic value of microRNA expression profiling in breast cancer has also been demonstrated by Iorio and colleagues [68]. A paper recently published in Cancer Research reported that microRNA signatures were superior in predicting overall survival in squamous cell carcinoma (SCC) patients than previous mRNA-based signatures. This research further highlights potential clinical value of these novel biomarkers in cancer prognosis [69].

## PERSPECTIVE

Studying the oncomirs is helpful in understanding the whole regulation network of initiation and progression of cancers, and new findings in the field could be promising ways in diagnosis, therapy and prognosis. However, treating cancers by taming these miRNAs need more efforts. The fundamental mechanisms require more detailed elaborations, target mRNAs have to be highly specific, and more effective small RNA delivery systems have to be introduced. Taken together, from lab bench to clinical bed, there is still a long way to go.

## Figures and Tables

**Fig. (1):**
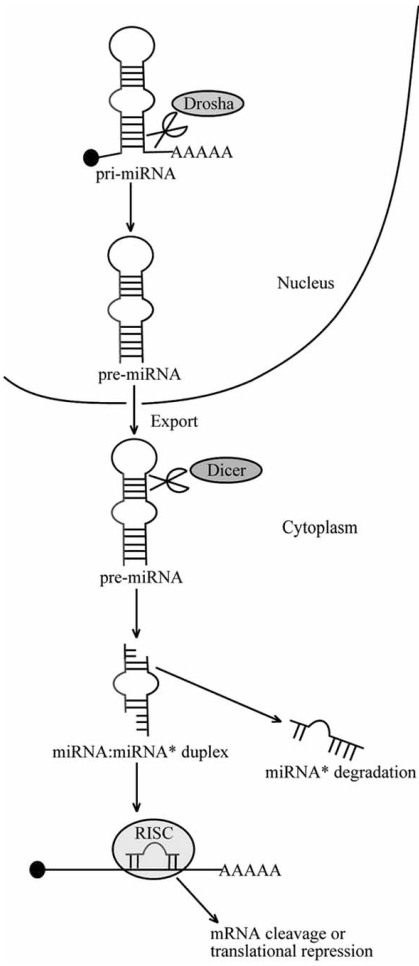
**Overview of microRNAs biogenesis.** MicroRNA is transcribed as pri-miRNA in nucleus and then processed into pre-miRNA by Drosha. After exportated to the cytoplasm, the pre-miRNA is processed by Dicer into a small dsRNA called the miRNA:miRNA* duplex. The active strand, which is the mature miRNA is incorporated into the RISC and binds to the target mRNA, whereas the inactive strand is ejected and degraded. The complementarity of the miRNA and the mRNA leads to translational inhibition or mRNA cleavage.

**Fig. (2).**
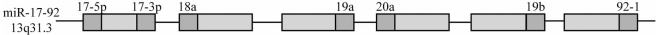
Genomic organization of the *miR-17-92* cluster.

**Fig. (3):**
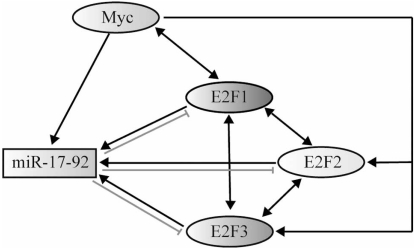
**The interactions among c-Myc, E2Fs and the *miR-17-92* cluster.** Arrows indicate induction of gene expression. Bidirectional arrows refer to mutual transcriptional induction. Hammer-heads indicate translational inhibition or degradation of mRNAs targeted by the *miR-17-92* cluster.

**Table 1:** Functions of Some Critical Targets of The *miR-17-92* Cluster

Name	Function
E2Fs	Positive regulators of cell proliferation, Proapoptotic proteins [42, 43]
CDKN1A (p21)	Negative regulator of G_1_-S checkpoint [46, 47]
BIM	Proapoptotic protein [46, 47]
Tsp1	Anti-angiogenic protein [49, 50]
CTGF	Anti-angiogenic protein [49, 50]
AIB1	Oncogenic protein [59]
Cyclin D1	Oncogenic protein [60]

## ABBREVIATIONS

miRNA: = microRNA
siRNA: = short interfering RNA
dsRNA: = double-stranded RNA
RISC: = RNA-induced silencing complex
Ago proteins: = Argonaute proteins
PTGS: = Posttranscriptional gene silencing
C13orf25: = Chromosome 13 open reading frame 25
DLBCL: = Diffuse large B-cell lymphoma
ChIP: = Chromatin immunoprecipitation
SCLC: = Small-cell lung cancer
Tsp1: = Thrombospondin-1
CTGF: = Connective tissue growth factor
AIB1: = Amplified in breast cancer 1
IPA: = Ingenuity pathways analysis
AMOs: = Anti-miRNA oligonucleotides
SCC: = Squamous cell carcinoma
